# TiO_2_ Nanoparticles and Their Effects on Eukaryotic Cells: A Double-Edged Sword

**DOI:** 10.3390/ijms232012353

**Published:** 2022-10-15

**Authors:** Jan Gojznikar, Bogdan Zdravković, Marko Vidak, Brane Leskošek, Polonca Ferk

**Affiliations:** 1 Institute for Biostatistics and Medical Informatics/ELIXIR-SI Center, Faculty of Medicine, University of Ljubljana, Karantanska ulica 37, 2000 Maribor, Slovenia; 2 Department of Anesthesiology, Intensive Care and Pain Management, University Medical Centre Maribor, Ljubljanska ulica 5, 2000 Maribor, Slovenia; 3 Institute for Biostatistics and Medical Informatics/ELIXIR-SI Center, Faculty of Medicine, University of Ljubljana, Vrazov trg 2, 1000 Ljubljana, Slovenia

**Keywords:** TiO_2_ NPs, eukaryotic cell, cytotoxicity, ROS, human health, aquatic organisms, agricultural plants, environment

## Abstract

Nanoparticulate TiO_2_ (TiO_2_ NPs) is a widely used material, whose potential toxicity towards eukaryotic cells has been addressed by multiple studies. TiO_2_ NPs are considered toxic due to their production of reactive oxygen species (ROS), which can, among others, lead to cellular damage, inflammatory responses, and differences in gene expression. TiO_2_ NPs exhibited toxicity in multiple organs in animals, generating potential health risks also in humans, such as developing tumors or progress of preexisting cancer processes. On the other hand, the capability of TiO_2_ NPs to induce cell death has found application in photodynamic therapy of cancers. In aquatic environments, much has been done in understanding the impact of TiO_2_ on bivalves, in which an effect on hemocytes, among others, is reported. Adversities are also reported from other aquatic organisms, including primary producers. These are affected also on land and though some potential benefit might exist when it comes to agricultural plants, TiO_2_ can also lead to cellular damage and should be considered when it comes to transfer along the food chain towards human consumers. In general, much work still needs to be done to unravel the delicate balance between beneficial and detrimental effects of TiO_2_ NPs on eukaryotic cells.

## 1. Introduction

Nanoparticulate titanium dioxide (TiO_2_ NP) is a metallic-oxide compound that is of wide use and interest in many aspects of modern society. Being semiconductive, it has high particle stability, coupled with low corrosivity and a purportedly lesser degree of toxicity than some other nanoparticles. It is also relatively cheap to produce. According to Noman et al. [1], three crystalline polymorphs exist—rutile, anatase and brookite, which differ in crystal structure. The former two possess a tetragonal structure, while the latter is rhombohedral [2]. Additionally, TiO_2_ NPs can also be found in amorphous forms [3].

A commonly noted property of TiO_2_ NPs is their photocatalytic ability, enabling them to stimulate generation of free radicals, which can then react further with other compounds [1,4]. Due to their photocatalytic and other properties, TiO_2_ NPs are widely used, including their application in solar cells. Other industrial uses of the compound include use in coatings, sensors and electronic devices, while probably one of the most known uses is the use of TiO_2_ NPs as a white pigment. The latter is applied in a broad spectrum, ranging from paints to cosmetic products, such as sunscreen or toothpaste [1,2,5]. Additionally, the material is even found in food products as an additive (E 171; [5,6,7,8]). Probably due to its wide applicability, TiO_2_ NPs are among the most produced nanomaterials in the world [9].

Apart from its industrial use due to favorable physical and chemical properties, TiO_2_ NPs can potentially even generate benefits linked to human health, such as cancer treatment [10,11,12]. Several studies also indicate its potential in the field of agriculture (e.g., [13,14,15,16]). On the other hand, TiO_2_ NPs can cause toxicological effects to eukaryotic cells (such as apoptosis and necrosis, see [17]), including potential risks to human well-being, as well as having detrimental environmental effects [6,18]. As put forward by Musial et al. [5] for the area of human consumption, inconsistencies in results can be found when examining various studies, dealing with the impact of TiO_2_ NPs.

This review is an attempt to summarize TiO_2_ NPs detrimental and possible beneficial effects in human and other eukaryotic cells, mainly with regards to human health and the environment.

## 2. Molecular Mechanisms of TiO_2_ Toxicity

**Figure 1:**
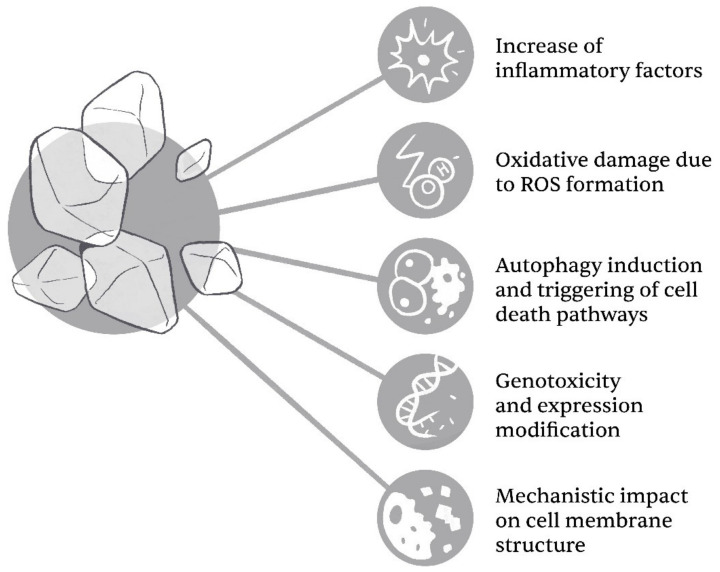
The general influences of cytotoxicity of TiO_2_ NPs on eukaryotic cells. Mechanisms, presented herein, are often interconnected (Illustrator: Matevž Bervar).

The cytotoxicity of TiO_2_ NPs is linked to both their size and form. Lu et al. [8] have recently demonstrated that the cytotoxicity of TiO_2_ seems to be inversely connected to their size—for example, by observing LDH leakage, where the author’s report an increase of the latter with a reduction of particle size. When it comes to form, both of the most common crystalline forms—anatase and rutile—seem to present cytotoxic risk, with varying results between studies [19,20,21]. The general cytotoxicity impact/mechanisms of TiO_2_ NPs on cellular damage are summarized in Figure 1.

TiO_2_ NPs induce inflammation by increasing the level of NF-κB (p65), HSP 60, p38 protein, IFN-γ, TNF-α, and nitric oxide, in a dose-dependent manner [22]. Some inflammation mediators induced by TiO_2_ NPs are organ-specific; for example, the brain inflammation markers IL-1β, IP-10 and CXCL1 [23]. Exposure of mice to ingested TiO_2_ NPs led to a stronger colonic inflammation than exposure to non-nTiO_2_ particles that are used in the food additive E171. TiO_2_ NPs significantly increased the colonic level of the pro-inflammatory cytokines IL-17 and KC/GRO, while TiO_2_ E171 showed no significant effects on these two cytokines. Exposure to ingested TiO_2_ NPs resulted in inflammation of the colonic mucosa, characterized by formation of dysplasia, distortion of crypts, widespread loss of goblet cells, and mucosal infiltration. However, both TiO_2_ NPs and TiO_2_ E171 decreased the colonic level of IL-10, an anti-inflammatory cytokine [24].

Formation of ROS is another important mechanism of toxicity of TiO_2_ NPs [25]. Exposure to TiO_2_ NPs following intranasal administration activates heme oxygenase 1 through the p38-Nrf-2 signaling pathway, leading to ROS accumulation and oxidation of lipids, proteins, and DNA. This mechanism triggered brain injury in mice [26]. Indirect DNA damage via oxidative stress results in frequent p53 mutations, elevated hepatic malondialdehyde (MDA-marker of lipid peroxidation), and decreased antioxidant defense systems. Administration of free radical scavengers such as chlorophyllin (CHL) alleviates the oxidative stress and reduces genotoxicity of TiO_2_ NPs [27]. H_2_O_2_ can be generated in photocatalytic reactions with TiO_2_ as the catalyst. H_2_O_2_ production is favored when TiO_2_ surface areas exceed 200 m2/g, as is the case for TiO_2_ NPs [28]. There are two mechanisms of H_2_O_2_ formation in TiO_2_ photocatalysis, and the mechanisms depend on the oxygenation rate of aqueous solutions. In oxygenated solutions, H_2_O_2_ is formed directly through reduction of molecular oxygen by conduction band electrons, while in oxygen-free solutions H_2_O_2_ can only be formed through recombination of hydroxyl radicals that originate from photocatalytic disintegration of water molecules. These mechanisms were confirmed by experiments using the radical scavenger tris(hydroxymethyl)aminomethane (Tris). H_2_O_2_ concentration increased in the oxygenated solution as Tris protected H_2_O_2_ from decomposition by hydroxyl radicals, while no H_2_O_2_ could be detected in the deoxygenated solution due to the scavenging of hydroxyl radicals by Tris, which prevented H_2_O_2_ formation [29].

There are several reports on how TiO_2_ NPs affect apoptosis, necrosis, necroptosis and cell cycle in human and other eukaryotic cells [17]. Astrocyte-like U87 cells treated with TiO_2_ NPs showed both Annexin-V staining, a marker of apoptosis, and propidium iodide (PI) staining, a marker of necrosis. Both types of the cellular staining were increased with the elevation in the treatment concentrations of TiO_2_ NPs [30]. Apoptosis induced by TiO_2_ NPs was also confirmed by measurements of mitochondrial membrane potential. Effects of TiO_2_ NPs on cell cycle are dependent on the crystalline phase of particles. Pure anatase TiO_2_ NPs arrested the cell cycle of neuronal cells at S-phase while only slight cell cycle changes were observed for the mix of anatase and rutile particles [19]. Necroptosis can also occur in lung cells exposed to TiO_2_ NPs. After the exposure of a primary culture of alveolar macrophages to TiO_2_ NPs, cell death increased in correlation with TiO_2_ concentration. In addition, the cytoplasm of alveolar macrophages exposed to TiO_2_ NPs showed expression of phosphorylated mixed lineage kinase (pMLKL) and high-mobility group box-1 (HMGB1), which are key indicators of necroptosis. Treatment of the exposed alveolar macrophages with Nec-1s (a necroptosis inhibitor) inhibited cell death [31].

TiO_2_ NPs-induced necroptotic pathways were also detected in A375 human melanoma cells. In smaller doses, the cells responded with a non-detrimental autophagy flux, whilst under the influence of photoactivation, autophagy is hindered, and a necroptotic reaction was triggered by production of ROS [32]. In addition, TiO_2_ NPs induce autophagy in normal lung cells. The extent of autophagy was determined quantitatively by the ratio of LC3 II to LC3 I, which increased significantly in cells treated with TiO_2_ NPs [33]. This finding is relevant since inhalation is a major absorption route for TiO_2_ NPs and lung cells represent the first line of contact with inhaled particles.

TiO_2_ NPs may cause toxic effects also through other mechanisms such as upregulation of tight junction proteins in the blood–brain barrier, as well as modulation of P-gp mRNA expression [23]. TiO_2_ NPs can also upregulate some enzymes; for example, alanine aminotransferase (ALT) and aspartate aminotransferase (AST) in the liver. Significant increases in ALT and AST were reported in mice [34], likely caused by the accumulation of TiO_2_ NPs in the liver. Furthermore, TiO_2_ NPs were reported to decrease the expression of dopamine receptors D1 and D2 in mice. Exposure to TiO_2_ NPs also decreased the expression of neurotrophins and related receptors, such as nerve growth factor (NGF) and its receptor TrkA, as well as brain-derived neurotrophic factor (BDNF) and its receptor TrkB [35].

Differential expression analysis of the entire proteome and phosphorylated proteome of primary macrophages showed that exposure to TiO_2_ NPs affected the expression of around 1000 proteins. TiO_2_ NPs had more impact on the phosphorylated proteins due to strong interaction of nanoparticles with phosphate groups in proteins. Differentially expressed proteins affected cellular component assembly, cell localization and growth, homeostasis, and tyrosine kinase signaling pathways. Differentially expressed phosphorylated proteins were involved in regulation of adaptive immune response, receptor mediated phagocytosis, and anion transmembrane transport. Exposure of macrophages to TiO_2_ NPs elicits inflammatory responses through up-regulation of tumour necrosis factor α (TNFα) and inducible nitric oxide synthase (iNOS). Metabolome analysis of macrophages revealed that TiO_2_ NPs activated the cyclooxygenase-2 (COX-2) pathway in macrophages. Quantification of metabolites derived from arachidonic acid detected a significant increase of the prostaglandins PGD2, PGE2, and 15d-PGJ2 [36].

TiO_2_ NPs that are orally ingested can adsorb proteins from food within the gastrointestinal tract. Formation of a protein corona around TiO_2_ NPs was confirmed for plant-based proteins glutenin, gliadin, zein, and soy protein that are common nutritional ingredients. A corona of adsorbed proteins can affect bioavailability of nanoparticles and the composition of the gut microbiota [37]. TiO_2_ particles that enter the bloodstream can adsorb serum proteins such as albumin and alter their physiological function [38].

Additionally, cytotoxic and genotoxic effects of TiO_2_ NPs on eukaryotic cells were observed. Hou et al. [25] highlighted the attachment of these large-surfaced particles onto cell membranes and other cellular structures, leading to cytotoxicity. Some form of cell membrane or cell wall damage might occur even when UV light is not present in conjunction with TiO_2_ [39], as, for example, what was observed in hamster ovary cells by Batiuskaite et al. [40]. Regarding genotoxicity of TiO_2_ NPs, many in vivo and in vitro studies were conducted, and overall, the studies indicating that TiO_2_ NPs are genotoxic outweigh the studies that state otherwise. Thus, TiO_2_ NPs can be earnestly treated as a potential health hazard [41].

## 3. TiO_2_ NPs Hazard for Human Health

**Figure 2:**
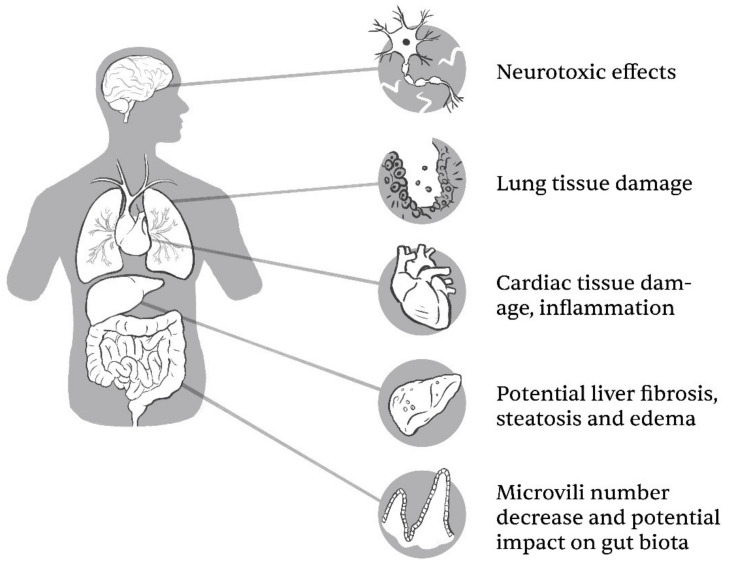
TiO_2_ NPs can potentially cause adverse effects on a cellular level in a variety of organ systems within the human body. Apart from nervous, respiratory, cardiovascular and digestive systems, there is some report of toxicity towards the skin and of potential impacts on embryonal development (Illustrator: Matevž Bervar).

Daily, humans are exposed to TiO_2_ NPs in various ways, mainly by inhalation in occupational settings, via certain consumers’ goods as food supplements (E 171), skin contact via cosmetical products and pharmaceuticals [7,42]; hence, their potential effect on the human body should not be overlooked. In vivo tests in animals revealed that inhalation, oral exposure, intraperitoneal and intravenous administration of TiO_2_ NPs can lead to absorption of particles in the bloodstream and accumulation of TiO_2_ in various organs, the liver, kidneys, spleen, lungs, brain, alimentary tract and heart, generating potential health risks (summarized in Figure 2) [42,43,44,45].

### 3.1. Effects of TiO_2_ NPs on Nervous System

To reach the brain, TiO_2_ NPs must cross the blood–brain barrier or migrate along afferent nerve fibers; both mechanisms have been described as feasible. Several in vivo and in vitro studies have demonstrated the neurotoxicity of TiO_2_ NPs through programmed cell death processes, such as apoptosis, autophagy and necroptosis depending on the duration of exposure and the dose-dependence. Although low-dose exposure could not induce any acute neurotoxicity, chronic exposure to low dose TiO_2_ NPs results in TiO_2_ accumulation and might lead to dramatic changes in the brain [43,46,47,48,49,50].

All the research objects in experiments so far only consisted of animals (such as mice and rats) or cells (such as PC12, U373, C6) [43]. In consequence, neurotoxic data of TiO_2_ NPs collected from those studies might be inappropriate to determine their neurotoxic effects on humans. To fully understand the neurotoxicity of TiO_2_ NPs, studies of human exposures to TiO_2_ NPs are needed [46].

### 3.2. Cardiovascular Effects of TiO_2_ NPs

Some studies showed that TiO_2_ NPs could be toxic and have detrimental effects on the cardiovascular system [43,50]. Rossi et al. [51] report that inhaled TiO_2_ NPs acutely increased cardiac excitability and promoted arrhythmogenesis in normotensive rats by a direct interaction with cardiac cells. Chen et al. [52], in their in vivo tests on rats, set forth a hypothesis that cardiac damage and inflammatory response caused by TiO_2_ NPs were suggested as the possible reasons for cardiovascular adverse effects. Zhao et al. [53] studied cardiopulmonary effects among workers who were exposed to TiO_2_ NPs. Occupational exposure by airborne TiO_2_ NPs was associated with oxidative stress, inflammation, lung and cardiac damage, indicated by the workers’ serum cardiopulmonary disease markers. Some workers were found to have symptoms such as shortness of breath and even suffered from pericardial effusions and hypoxemia [49]. However, most of the doses employed in studies are too high to be realistic in occupational settings [41].

### 3.3. Effects of TiO_2_ NPs on Pulmonary System

Pulmonary in vivo studies on rats support the carcinogenicity of TiO_2_ NPs in intratracheal and inhalation studies [41]. Mitochondrial damage, oxidative stress, and apoptotic cell death in a dose-dependent manner were involved in the toxic mechanistic pathways [47]. Lung inflammation was associated with dose-dependent increases. However, no clinical signs of obvious acute toxicity were observed [41,47]. In vitro, cell lines of human lung origin showed increased ROS generation and apoptotic death on exposure to TiO_2_ NPs [54]. Human lung damage (functional loss, elevated markers of oxidative stress, and inflammation) on exposure to airborne TiO_2_ NP has been reported [53]. However, the results from a few epidemiological studies show that there are no significant associations between TiO_2_ NPs exposure and risk of lung cancer. The relatively short history in production and use seems to be the main reason for the lack of human epidemiological studies for TiO_2_ NPs. Epidemiological studies thus far have not been able to detect an association between the occupational exposure to TiO_2_ NPs and an increased risk for cancer [41,55].

### 3.4. Effects of TiO_2_ NPs on Liver, Spleen and Kidneys

Mice were treated with different dosing regimens of TiO_2_ NPs injected intraperitoneally. In general, high doses of TiO_2_ NPs significantly increased the organ/body weight ratio of the liver, spleen and kidneys in mice in a dose-dependent manner [43] Intravenously applied TiO_2_ NPs caused massive oxidative stress, DNA damage, and mitochondria-mediated apoptosis in the kidney and liver of rats [47]. Other observations included elevated liver function biomarkers, necrosis of liver cells and apoptosis, liver fibrosis and swelling of renal glomeruli in high doses of TiO_2_ NPs [43,50].

As is argued in Heringa et al. (2018) and Peters et al. (2020) see [42], it can be assumed that TiO_2_ found in human tissues, in individuals without an occupational inhalatory exposure, almost exclusively originates from oral exposure to TiO_2_ particles. Analysis of postmortem tissues indicates that these particles are taken up by the intestine and subsequently transported to secondary organs such as liver, spleen and kidneys, where they can accumulate. A number of in vivo toxicity studies on rats/mice show that TiO_2_ NPs can trigger ROS generation, induce oxidative stress, inflammation and might lead to liver fibrosis, steatosis and edema, but it is unknown whether these events subsequently result in irreversible adverse effects in humans. With regard to kidneys and spleen, data are too limited to draw conclusions on potential health risks in humans. The level presence of TiO_2_ in these organs indicates that it is relevant to gain further knowledge on potential effects of TiO_2_ on these tissues [42].

### 3.5. Effects of TiO_2_ NPs on Alimentary Tract

The results of recent studies on rats and the human volunteer showed that TiO_2_ NPs were scarcely captured from the GIT and transferred into systemic circulation [43]. Available data suggest that TiO_2_ NPs may influence microbiota, disturb digestion and absorption of food components causing problems such as inflammatory bowel disease, obesity, or deficiencies of macro- and microelements in the body. Indeed, there are increasing concerns that its presence may lead to intestinal barrier impairment, including dysbiosis of intestinal microbiota [43,56]. Studies concerning toxicity after oral administration to rats show no obvious signs of acute toxicity [41,43]. Duan et al. demonstrated weight loss in mice after intragastric administration of TiO_2_ NPs. This should be explained by the reduced number of intestinal villi and the resulting loss of surface of the small intestine capable of absorbing nutrients, which consequently leads to malnutrition and weight loss [43]. Amedollia et al. also showed in their studies that after oral exposure of rats, TiO_2_ NPs are capable of penetrating the intestinal mucosa. The results of in vivo tests confirmed the results of in vitro studies on human intestine cell cultures. They found a decrease in the number of microvilli, resulting in a reduction of the surface area available for absorption of nutrients [43].

At the same time, there have been studies that revealed a limited influence of TiO_2_ NPs on human microbiota. Currently available reports therefore provide contradictory evidence in terms of the impact of TiO_2_ NPs on human microbiota due to the application of varying experimental models and frameworks [56].

### 3.6. Skin Toxicity of TiO_2_ NPs

Inclusion of TiO_2_ NPs into sunscreens has raised interesting questions regarding the potential for dermal penetration, systemic absorption, and subsequent toxicity. The in vitro and in vivo data regarding the potential of dermal absorption and/or penetration of TiO_2_ NPs from sunscreens exhibit controversial results. Although several articles describe the opposite, the penetration of TiO_2_ NPs in healthy, as well as in damaged or lesioned skin, is demonstrated in the scientific community, with damaged or lesioned skin being more susceptible to TiO_2_ NPs penetration. The fact that upon skin penetration, TiO_2_ NPs can enter the blood circulation, and then undergo translocation to various distant tissues and organs, suggests that prolonged time exposure to TiO_2_ NPs, may pose a health risk to consumers [57].

Extensive studies have shown that TiO_2_ NPs can cause cell toxicity under in vitro and in vivo conditions. However, cosmetic industries are using alternative methods to animal experimentation, such as 3D skin reconstructed models, to evaluate the irritation and corrosion of cosmetics. Sanches et al. [57] have shown that the 3D skin model can be used to test the toxicity of TiO_2_ NPs. The data obtained show that TiO_2_ NPs was considered non-irritant, non-corrosive and non-phototoxic for the 3D skin model, regardless of the crystalline type and the size of the NPs studied [57].

Melanoma is the malignancy responsible for the highest incidence of deaths from skin cancer. As is argued in Zdravković et al. [58], it can be assumed that TiO_2_ NPs may even influence metastatic melanoma cells’ invasiveness and aggressiveness. In practice, results obtained from the study suggest that patients with diagnosed metastatic melanoma should avoid the usage of products containing TiO_2_ NPs because this could increase progression and recurrence of their disease [58].

### 3.7. Embryotoxicity of TiO_2_ NPs

A great number of in vivo studies on pregnant mice/rats exposed to TiO_2_ NPs by various routes, including intraperitoneal and intravenous injection, revealed that those NPs could be detected in the brain of the fetus, induce an acute stress reaction on the neurons and glial cells of mouse brains and lead to CNS dysfunction, as a consequence. The authors discovered that they can even cause pregnancy complications, increase dopamine levels in the prefrontal cortex and neostriatum, alter gene expression related to brain development and enhance depressive-like behaviors in mice/rats. All the data collected from in vivo and ex vivo mouse embryo studies showed that they exhibited toxic effects on the growth and development in dose-dependent and time-dependent manners. Stemming from all above, they may affect the brain development of embryos by crossing the placental barrier and lead to CNS dysfunction later in life [46,50].

### 3.8. Potential Clinical Use of TiO_2_ NPs

Although numerous in vivo and in vitro studies have provided evidence of the toxic effects of TiO_2_ NPs, the capability to produce ROS that initiate programmed death has found application of targeted cytotoxicity of TiO_2_ NPs for photodynamic therapy (PDT) of cancers [10,11,12]. It was reported that modification of TiO_2_ NPs with some biomolecules that can specifically bind to cancer cells improved selectivity and efficiency of TiO_2_ to destroy cancer cells [59]. Wang et al. [60] report that combined TiO_2_ NPs exposure and UVA treatment in glioma-bearing mice led to a significant increase in the extent of necrotic areas and indices of apoptosis in established tumor grafts. Moreover, it is obvious that there is a synergistic effect of UV irradiation and TiO_2_ NPs on the drug uptake of target leukemia cells, which may also be utilized as a new strategy to inhibit the drug resistance of the targeted cancer cells, suggesting the feasible idea of cancer treatment using TiO_2_ NPs and UV irradiation with accompanying anticancer drugs [61]. Cai et al. [62] reported that photoexcited TiO_2_ particles effectively induced cytotoxicity against HeLa cancer cells. Some research illustrated that human colon carcinoma cells treated with photoexcited TiO_2_ NPs can be effectively affected and damaged [63]. Experimental exposure of TiO_2_ NPs was found to induce oxidative stress, genotoxicity, and apoptosis in human lung cancer cell line A549 [64].

## 4. TiO_2_ NPs and Other Eukaryotes—Toxic or Not?

The impact of TiO_2_ NPs on eukaryotic cells expanding far beyond human cells [25] and the flow of material within ecosystems, alongside TiO_2_′s ever growing application, makes it an important potential toxicant. Ranging from small, superficially simple unicellular algae to complex organisms such as vertebrates, this section will therefore strive to provide the reader with an insight into the impact that TiO_2_ (especially in its nanoparticulate forms) has on other eukaryotes, based on a few randomly selected recent studies. Since plenty of review literature exists, concerning environmental/ecological aspects of nanoparticulate TiO_2_ [18,25,65,66,67], we strive to provide only a brief insight.

The environmental impact of this nanoparticulate metal oxide should not be underestimated, not only because it can have an adverse effect on single organisms and their ecosystem functioning, but also because its potential bioaccumulation, biomagnification and transfer through the food chain may very well, in the end, impact multiple species [20,66,68,69]. The complex interconnectivity between different trophic levels within and, indeed, between different ecosystems means that next to none are immune to TiO_2_ contamination. TiO_2_ NPs enter natural environments across the globe via direct application (in, e.g., agriculture), waste and residual materials, stemming from its growing use in industrial processes. As already stated, TiO_2_ is a known inductor of ROS production [70,71,72,73] and, in general, the authors of various research seem to agree that the effect of TiO_2_ is dependent foremost on its concentration/dosage and time of exposure. We can take a good example from mice (*Mus musculus*) where Liu et al. [74] have reported observable organ damage after exposing mice to multiple higher doses of TiO_2_ in nano-anatase form. Toxicity of TiO_2_ NPs may be enhanced with the presence of UV light [70,71,75,76,77]. Additional factors that one needs to consider is the form in which TiO_2_ is dosed—bulk or nanoparticle [70,73,78], although some research seems to dispute the differential impact of separate phases [79]. The mineral form also appears to be of importance (anatase/rutile; e.g., [20]) and it is also clear that TiO_2_ interacts with other nanoparticles and pollutants, potentially increasing or even decreasing their toxicity by means of potentially absorbing them on the surface of TiO_2_ particles [80,81,82,83,84,85,86].

### 4.1. TiO_2_ NPs and Their Effect on Aquatic Organisms

Prone to form agglomerates/aggregates in water-based media [70,71,79,87,88,89,90,91,92], TiO_2_ NPs are the focus of many studies, especially in aquatic environments, where nanoparticles are indeed of general concern [18,67,93,94,95]. Additionally, the material’s agglomeration can remove microorganisms from the water column [79]. The actual effect of TiO_2_ remains disputed, with Dedman et al. [79] even suggesting that its effect at environmentally realistic concentrations might be disregarded under certain circumstances, at least on a microbial level, challenging the view of, e.g., Miller et al. [75], who find that TiO_2_ is toxic to phytoplankton even in small quantities, when exposed to UV. Another study highlights that TiO_2_ under UV(A) radiation does not only impact organisms themselves, but also affects extracellular enzymatic activity in heterotrophic biofilms, therefore potentially affecting the transfer of matter and energy in natural aquatic ecosystems [96].

As primary producers, aquatic plants and algae are of great importance. When exploring the effect of TiO_2_ (both in bulk and nanoparticulate form) whilst under exposure to visible or UV-A light, two microalgae species, *Chlamydomonas reinhardtii* (freshwater) and *Phaeodactylum tricornutum* (seawater)), reported signs of toxicity (such as membrane damage), with nanoparticles producing significantly higher ROS content. High concentrations of TiO_2_ also stimulated the production of exo-polymeric substances, which could be connected to a defensive response, yet their role is not entirely understood [70]. Negative toxic effects were reported also for the diatom *Thalassiosira pseudonana* [97], green alga *Dunaliella tertiolecta* [98] and *Chlorella vulgaris* [99]. In the latter, another environmental concern comes into effect, since ocean acidification increased nanoparticle internalization [99]. Thiagarajan et al. [76] explored the difference in sensitivity between *D. salina* and *Chlorella* sp. (the latter being more sensitive), noting a decrease in cell viability, an increase in ROS production, and lipid peroxidation. In the duckmeat species *Spirodela polyrhiza*, Movafeghi et al. [72] report an observable uptake into plant tissues/cells, which apparently resulted in a reduction of growth and photosynthetic pigment content in connection to concentration

Moving further upwards the food chain, a multitude of studies has focused especially on shellfish, since these organisms are often of commercial interest. Among them, bivalves (Phylum Mollusca, Class Bivalvia) have garnered considerable attention [95] since they mainly feed by filtering water and sediment and are therefore highly prone to intake of any suspended nanoparticles from the water column [88]. In the Mediterranean mussel (*Mytilus galloprovincialis*), a species often consumed as a food item across the Mediterranean Basin, TiO_2_ NPs can apparently decrease lysosomal membrane stability and alter the activity of antioxidant enzymes in the mussel’s digestive gland. Although no changes in morphology were observed, nanoparticles were noted inside lysosomal vacuoles of the cells. Decreased lysosomal membrane stability, alongside a decrease in phagocytosis was also observed in hemocytes (which are the immune cells of invertebrates) [80,89]. Phagocytosis is usually seen as an important part of immune defense in bivalves and an effect on hemocytes could potentially mean a more readily available transfer of pathogens toward human consumers [88]. Some increase in nitrite accumulation, changes in immune-related gene expression and production of extracellular ROS, DNA damage (at a chromosomal level) and potential pre-apoptotic processes (nuclear abnormalities) were also noted [80,87,89]. In general, TiO_2_ NPs do not seem to exhibit substantial cytotoxic effects but do have some role in triggering immune and inflammatory pathways [87]. The reduction of phagocytic activity was also observed in the Eastern oyster (*Crassostrea virginica*) [88] and an effect on hemocytes was also reported from three Pacific bivalve species-the Gray’s mussel *Crenomytilus grayanus*, the horse mussel *Modiolus modiolus* and *Arca boucardi*. TiO_2_ induced mortality and changes in membrane polarization in hemocytes, although with considerable variation among species [100].

In *M. galloprovincialis*, Canesi et al. [80] also report both antagonistic and synergistic effects of TiO_2_ and 2,3,7,8-tetrachlorodibenzo-p-dioxins (TCDD), depending on the cell/tissue type and molecular aspect examined. For example, an increased lysosome to cytoplasm ratio was observed when exposed to both contaminants, with the author’s highlighting that larger concentrations of bioaccumulated TCDD in the presence of TiO_2_ might represent a Trojan horse effect. The latter effect was also observed in another bivalve species *Corbicula fluminea* [82] and Shi et al. [84] report an increase in immunotoxicity towards hemocytes of the blood clam *Tegillarca granosa* due to a potential Trojan horse effect of TiO_2_ on 17β-estradiol (E2). TiO_2_ alone did not impact total hemocyte count of *T. granosa*, but hindered phagocytotic rates after 10 days of exposure. The authors also report a significant reduction of expression in tested genes, connected to the immune ability of the clam [84].

Other aquatic animals have also been explored, ranging from crustaceans [20,77], through sea urchins [101], to fish [86,90,102,103]. While testing the impact of multiple nanoparticles on *Strongylocentrotus intermedius* sea urchin for the purpose of using it in toxicity tests, Pikula et al. [101] noted no decrease in spermatozoan activity and only a low impact on egg fertilization. Additionally, TiO_2_ was among the lowest acutely toxic nanomaterials when it came to the mortality of embryos; this, however, changed by passing time, since the authors noted a time-dependent increase in toxicity. It is also very interesting to note that TiO_2_ NPs showed no apparent toxicity on brine shrimp *Artemia salina*, even under high concentration. Nonetheless, this metal oxide did have some role in the degree of toxicity of other pollutants, such as cadmium and phenanthrene [83]. Under certain circumstances, TiO_2_ was reported to be harmful to *Daphnia magna*, where TiO_2_ NPs in conjunction with UV light upscaled the production of the toxic -OH radical [77]. In *D. magna*, an impact on the digestive tract was also observed, e.g., with digestive cells producing noticeable autophagy vacuoles [20].

Fish are especially interesting when it comes to potential TiO_2_ NPs effects, as they often represent the higher rungs of the aquatic food chain. Consumption of prey organisms can lead to bioaccumulation as observed in turbots (*Scophthalmus maximus*), fed with TiO_2_ “contaminated” clamworms (*Perinereis aibuhitensis*). Such consumption among others induced tissue abnormalities, such as liver fibrosis and splenocyte necrosis after a certain time period [68]. In zebrafish (*Danio rerio*), a significant decrease of leukocyte count 14 days after exposure, alongside an increase in glutathione levels, when exposed to TiO_2_ NPs, was observed by Ramsden et al. [90]. No apparent effect on the tissue morphology was noted [90]. Zeumer et al. [103], working with rainbow trout (*Oncorhynchus mykiss*), report an increase in acetylcholinesterase activity under exposure to wastewater treatment plant effluent supplemented with TiO_2_ NPs, indicating potential chronic neurotoxic effects of TiO_2_ in conjunction with organic matter. Synergistic effects of TiO_2_ and other nanoparticles in fish are also reported. When co-examined with lead (Pb), TiO_2_ increased its toxicity and induced several changes, including gene expression, in *D. rerio* embryos [85]. In the common carp (*Cyprinus carpio*), the role of TiO_2_ NPs in enhancing acute toxicity of silver nanoparticles has been observed by Haghighat et al. [86]. The addition of only TiO_2_ showed less of an impact, yet nonetheless stimulated some deformities in gill tissue, including an increase in cellular proliferation (defined as hyperplasia of chloride cells [86]). A variation of chloride cell count, alongside goblet cells, was also observed, probably as a protective response, when exposing gills of the Senegalese sole (*Solea senegalensis*) to TiO_2_ NPs. Additionally, gill macrophages contained observable black metal deposits after two different TiO_2_ treatments [102].

Not even sea mammals are immune to the effects of TiO_2_ NPs. Bernardeschi et al. [104] report that both anatase and rutile form present a potential toxic element to a widespread sea mammal, the bottlenose dolphin (*Tursiops truncatus*). The authors noted an increase in DNA fragmentation in dolphin leukocytes, yet nonetheless note that this preliminarily observed effect seems to occur to a lesser degree when compared to human leukocytes at equal concentrations of TiO_2_ [104].

**Figure 3:**
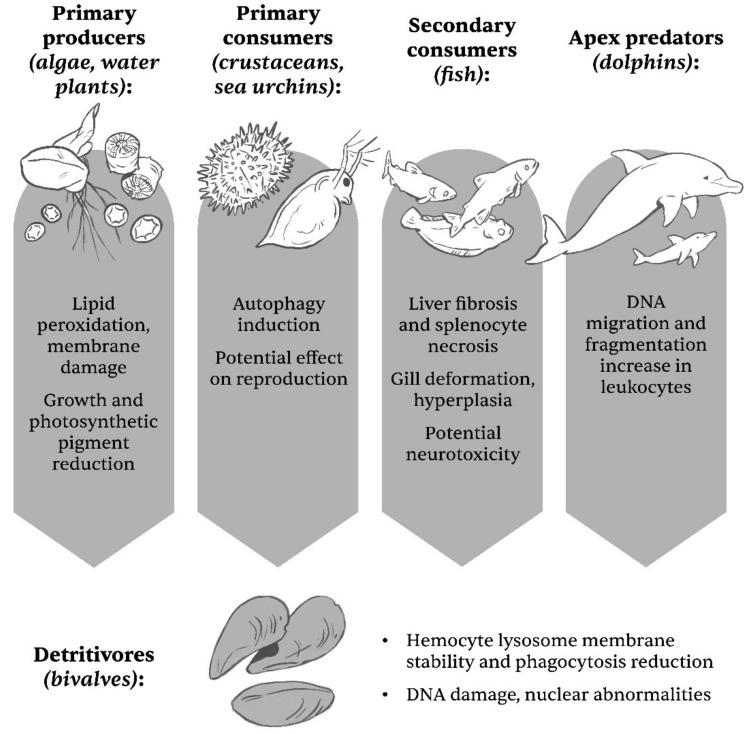
Some of the effects of TiO_2_ NPs on various organisms consisting different trophic levels in aquatic ecosystems. Apart from bioaccumulation and biomagnification, one should not ignore the impact of potential biomass reduction from one of the rungs due to TiO_2_ NPs toxicity, which could in turn affect all other levels (Illustrator: Matevž Bervar).

In summary, TiO_2_ NPs affect various trophic levels in aquatic biosystems, which is illustrated in Figure 3. The potential of transfer and potential bioaccumulation/magnification [20,68] should therefore not be underestimated.

### 4.2. TiO_2_ NPs and Agricultural Plants

The impact of TiO_2_ is not limited only to aquatic vegetation and its toxic effects have also been explored in terrestrial plants, where accumulation/uptake appears to occur via their root system in a concentration dependent manner [13,69,92]. Although detrimental effects of TiO_2_ should always be of concern (e.g., [73]), it is apparent that TiO_2_ can induce some changes that could be considered beneficial, such as an increase in chlorophyll content and connected plant weight/growth, at least at lower concentrations [13,14,15,16,105], which is particularly interesting in agriculture. However, using nanoparticles as fitomediatory means is a double-edged sword, since dietary uptake into human consumers can follow, generating health risks [13].

In rice (*Oryza sativa*), an increase of hydrogen peroxide (H_2_O_2_) production, membrane injury and rate of root cell death was observed at higher concentrations, likely connected to an increase in oxidative stress [15]. On the other hand, TiO_2_ NPs seemingly boosted chlorophyll content, biomass, and increased phosphorus and protein grain content [14,15], even when another toxicant such as cadmium was present [106]. The after-expositional increase in rice biomass and photosynthetic activity was also reported by, e.g., Zhang et al. [107]. A similar effect was observed in maize (*Zea mays*), where treatment of waste waters with TiO_2_ was explored, with Yaqoob et al. [108] reporting positive effects on plant weight and photosynthetic pigments at lower concentrations of particles. The effect of concentration was also evident when studying another very common grain plant, wheat (*Triticum aestivum*), where Rafique et al. [109] observed an increase of H_2_O_2_ synthesis and formation of micronuclei with increased concentration. A very similar effect to that in rice (see [15]) was noted as well, with smaller dosages of TiO_2_ raising protein and nutrient content and biomass production, alongside an increase of H_2_O_2_ production [110]. A cytotoxic effect (measured by lactate dehydrogenase leakage), lipid peroxidation and imbalance in antioxidative enzyme activity were observed in *T. aestivum* calli cells by Czyżowska and Barbasz [111].

Moving from agriculturally important grasses (fam. Poaceae) to legumes (fam. Fabaceae), TiO_2_ NPs showed a clear capability to cause genotoxicity in the broad bean (*Vicia faba).* Although it had no noticeable effect on the mitotic index, it induced chromosomal aberrations, such as breaks. Additionally, it shortened shoot length. Both of these effects were apparently concentration-independent and when compared with SiO_2_ nanoparticles, TiO_2_ NPs seem to pose a higher cytotoxic risk [112]. A similar effect was also observed in the lentil (*Lens culinaris*), where TiO_2_ NPs in anatase form caused chromosomal DNA damage and mitotic index reduction in a concentration dependent manner, with other changes signaling excessive oxidative stress. Lower concentrations, however, seemed to present a moderate, yet significantly positive effect. Additionally, the nanoparticles reduced chlorophyll content [73]. On the contrary, no adverse effects were reported for TiO_2_ for the Adzuki bean (*Vigna angularis*); however, one should among others note that particles used by Jahan et al. [92] were larger than those used in *L. culinaris* by Khan et al. [73], which could play a role in differential response, as TiO_2_ NPs were demonstrated to be more toxic when particles are smaller [8]. In *V. angularis*, the metal oxide improved germination (though not when combined with ZnO), stimulated growth and increased chlorophyll content. Lack of any apparent stress in the plant in question was also confirmed by no significant change in antioxidant enzymatic stress activity. Coexistence of TiO_2_ and ZnO apparently also reduced the uptake of both nanoparticle species into the plant [92].

Spice plant cells are no exception when it comes to (potential) effects of TiO_2_ NPs. Significant research was done in fenugreek (*Trigonella foenum-graecum*) by Missaoui et al. [16,91,113], where nanoparticles seem to induce changes in mineral/metal content, hinting at root cell membrane damage [91]. In higher doses, a chlorosis of leaves due to decrease in chlorophyll a and b had also been observed, yet interestingly half of that concentration actually elevated the chlorophyll level. The same effect was observed when examining carotenoid content, which indicates that TiO_2_ NPs could affect photosynthetic ability of plant cells [16]. As with other plant species, fenugreek seems to be tolerant of lower concentrations of TiO_2_ NPs, balancing ROS production by increased antioxidant activity. Larger concentrations diminished defensive activity and therefore presented themselves as toxic to the plant [16,113]. Similarly, at a smaller degree, TiO_2_ seems to boost nutrient accumulation in coriander (*Coriandrum sativum*), yet larger concentrations show a potentially adverse effect, apparent in the activation of oxidative stress response and wrinkling of the cell membrane [114].

A limited effect was observed when studying the effect of TiO_2_ on the onion (*Allium cepa*), mainly observed as slight alterations to the mitotic index [115]. Singh and Kumar [13] report an increase in chlorophyll content in spinach (*Spinacia oleracea*) after treatment with sewage sludge containing TiO_2_ NPs. Gordillo–Delgrado et al. [105] also report that at certain small concentrations TiO_2_ can be beneficial to *S. oleracea*, even when combined with silver nanoparticles and could be of potential agronomic use.

## 5. Conclusions and Perspectives

It is possible that human exposure to TiO_2_ NP, whether related to occupational settings, environmental pollution, or certain consumers’ goods, may contribute to aggravation of several chronic diseases of humans and hence an increase in risk of developing tumors or progress of preexisting cancer processes. To support this, we can list the recent Commission Regulation (EU) 2022/63 (Official Journal of the European Union, L11/1, 18 January 2022), which has withdrawn TiO_2_ (E 171) as a food additive due to safety concerns. On the contrary, numerous studies enhance our understanding on cellular and molecular behavior of human cancer cells against TiO_2_ NPs exposure, which reveal excellent photochemical properties and hence show their potential use in new treatment modalities of malignant tumors. Extensive studies in this area are urgently needed.

Additionally, it is clear that TiO_2_ does indeed possess some qualities that, under certain circumstances, make it a viable hazard to eukaryotic cells across many organismal groups. As a widely present environmental contaminant, this metal oxide can induce oxidative stress and hinder cellular function. The impact of contamination should not be underestimated, with many studies highlighting the potential of TiO_2_ NPs to cause environmental damage. It would seem that special interest should be pointed towards aquatic ecosystems, where TiO_2_ is a common pollutant, with its effects becoming more and more well studied. Nonetheless, additional work is still needed to transplant research in situ, in the actual natural environment or at least one resembling it, where multiple factors need to be considered in addition to just the presence of TiO_2_. Further studies, following the behavior of particles along the trophic chain at a longer time period of exposure are therefore certainly required.

In conclusion, TiO_2_ clearly possess several attributes, that can either produce benefits or adversities when it comes to eukaryotic cells; however, much work is still needed to examine its potential impact. Future insights should also strive to unravel the delicate influence of dosage. Even though some benefit can be observed with smaller concentrations in the field of agriculture, we wish to conclude this review with the quote of the immortal Paracelsus: *Sola dosis facit venenum.*

## Data Availability

Not applicable.
